# Metal(loid) Concentrations in Coastal (*Sotalia guianensis*) and Oceanic (*Pseudorca crassidens*) Odontocete Cetaceans Stranded Along the Northeastern Brazilian Equatorial Margin

**DOI:** 10.1007/s12011-026-05137-y

**Published:** 2026-05-05

**Authors:** Rysónely Maclay de Oliveira, Radan Elvis Matias de Oliveira, Rachel Ann Hauser-Davis, Fernanda Loffler Niemayer Attademo, José Lailson Brito, Bárbara Manhães Moura Reis, Ana Bernadete Lima Fragoso, Flávio José de Lima Silva

**Affiliations:** 1 Programa de Pós-Graduação em Ciências Naturais, Universidade do Estado do Rio Grande do Norte, Mossoró, RN 59064-741 Brazil; 2 Programa de Pós-Graduação em Ciência Animal, Laboratório de Morfofisiologia Animal Aplicada (LABMORFA), Universidade Federal Rural do Semi- Árido, Mossoró, RN 59625- 900 Brazil; 3 Projeto Cetáceos da Costa Branca da Universidade Estadual do Rio Grande do Norte, Mossoró, RN 59610-210 Brazil; 4 Centro de Estudos e Monitoramento Ambiental, Areia Branca, RN 59655-000 Brazil; 5 Programa de Pós-Graduação em Ciência Animal, Universidade Federal da Paraíba, Areia, PB 58397-000 Brazil; 6 Laboratório de Avaliação e Promoção da Saúde Ambiental, Instituto Oswaldo Cruz, Fundação Oswaldo Cruz, Rio de Janeiro, RJ 21040-360 Brazil; 7 Laboratório de Mamíferos Aquáticos e Bioindicadores (MAQUA), Faculdade de Oceanografia, Universidade do Estado do Rio de Janeiro, Rio de Janeiro, RJ 20550-013 Brazil; 8 Programa de Pós-Graduação em Desenvolvimento e Meio Ambiente (PRODEMA), Universidade Federal do Rio Grande do Norte (UFRN), Natal, Rio Grande do Norte 59064- 741 Brazil; 9 Projeto Golfinho Rotador, Arquipélago de Fernando de Noronha, Pernambuco, 53990-000 Brazil

**Keywords:** Bioaccumulation, Ecotoxicology, Elemental contamination, Habitat, Marine mammals

## Abstract

Cetaceans face multiple threats, and pollution has become a major concern for their conservation worldwide. This study assessed metal(loid) concentrations in the tissues of two odontocete species with coastal and oceanic habits, namely Guiana dolphin (*Sotalia guianensis*; *n* = 11) and the false killer whale (*Pseudorca crassidens*; *n* = 8), respectively, stranded along the northeastern Brazilian equatorial margin. Silver (Ag), copper (Cu), cadmium (Cd), mercury (Hg), and selenium (Se) were determined in samples of liver, muscle, and kidney, expressed as mean ± standard deviation (SD), in µg g⁻¹ wet weight. In *P. crassidens*, Hg was detected in all individuals, with hepatic concentrations (794.1 ± 556.9 µg g⁻¹) higher than those in muscle (30.2 ± 16.7 µg g⁻¹); females exhibited higher levels than males in both tissues. In *S. guianensis*, mean Hg levels were also higher in the liver (1.6 ± 2.4 µg g⁻¹) compared to muscle (0.4 ± 0.4 µg g⁻¹), with the highest concentration detected in the kidney (3.1 µg g⁻¹). Detectable Cd concentrations occurred only in one juvenile (0.3 µg g⁻¹, liver). For Cu, Ag, and Se, values varied between species and tissues. The observed differences between species, as well as among sexes, age classes, and tissues, suggest that physiological factors, diet, and exposure time influence metal accumulation. These findings highlight the importance of considering the role of coastal versus oceanic habitat use, trophic ecology, and life history in the bioaccumulation of metal(loid)s since some of these factors were not assessed (i.e., trophic ecology).

**Supplementary Information:**

The online version contains supplementary material available at 10.1007/s12011-026-05137-y.

## Introduction

Odontocete cetaceans (“toothed whales”; order Cetacea; suborder Odontoceti) play an essential role in maintaining the ecological balance of marine environments, comprising top-predators [1, 2] and serving as important ocean ecosystem health sentinels [3–6]. Their wide geographic distribution, longevity, and high trophic position, however, make them particularly vulnerable to environmental pollutant bioaccumulation, such as metals and metalloids, hereafter termed metal(loids). Their presence in the globe’s oceans has increased substantially as a result of anthropogenic activities [7, 8]. Once introduced into aquatic ecosystems, mainly through industrial discharges, domestic sewage, mining, port activities, and soil leaching processes [8–10], these elements can accumulate in marine organisms and, in some cases, be transferred along the food chain [3, 7, 8]. Metal(loid) pollution, in fact, represents one of the main current marine fauna threats [8, 11], posing toxicological risks to exposed fauna, mainly by inducing oxidative stress, which may impair protein function, damage DNA, and disrupt membrane lipids [8, 9, 12–14]. Therefore, neurological, immunological, and renal alterations are detected, as well as decreased immunity and increased parasitic infestations [8, 10, 15, 16].

In this context, assessing metal(loid) levels in free-ranging odontocete species is essential for establishing species- and region-specific reference databases, enabling comparisons and improving our understanding of the impact of exposure to these contaminants on the health and survival of these animals. Moreover, the concentrations of these contaminants in tissues can vary according to several factors, including sex, age, trophic position, and geographic location [11]. In this context, continuous cetacean population monitoring concerning exposure to these toxicants is crucial.

Cetacean strandings have been frequently recorded in Brazil, particularly along the Northeastern coast. Within this area, the Guiana dolphin (*Sotalia guianensis*) and false killer whale (*Pseudorca crassidens*) are commonly reported [17, 18]. Strandings provide a unique opportunity to obtain biological and toxicological data from these animals, contributing to the understanding of the threats they face in the environment [19, 20]. Although the Potiguar Basin, in the Brazilian equatorial margin, is an important region for investigating these events, due to its strategic location and high biodiversity [21, 22], studies on metal(loid) levels in species stranded in this area are still scarce, with available information largely restricted to a single study conducted in southeastern Brazil [19].

*S. guianensis* occurs in shallow tropical and subtropical waters, primarily along the continental shelf of Central and South America, with a preference for estuaries, bays, and sheltered coastal areas [23, 24]. Its diet is predominantly composed of teleost fish, particularly from the Sciaenidae and Mugilidae families, followed by cephalopods (i.e., squid), and crustaceans. It also exhibits opportunistic feeding behavior, consuming seasonally available abundant prey, with variations depending on location and time of year [25–27]. In contrast, *P. crassidens* is a large odontocete cetacean, known for its complex social behavior, varied diet, and wide distribution in tropical and temperate waters, typically associated with oceanic environments or the deeper continental shelf [28, 29]. Its diet consists mainly of large fish and squid, and it exhibits cooperative hunting behavior, occasionally preying on other cetaceans [30–32].

The analysis of tissues pertaining to species with differing habitat-use patterns and feeding strategies can provide valuable insights into the ecological influence on metal(loid) contamination in odontocetes. Therefore, the present study aimed to analyze total mercury (Hg), copper (Cu), cadmium (Cd), silver (Ag), and selenium (Se) concentrations in the tissues of two odontocete species with coastal and oceanic habits, *S. guianensis* and *P. crassidens*, respectively, stranded along the Potiguar Basin.

## Material and Methods

### Sample Data

**Fig. 1:**
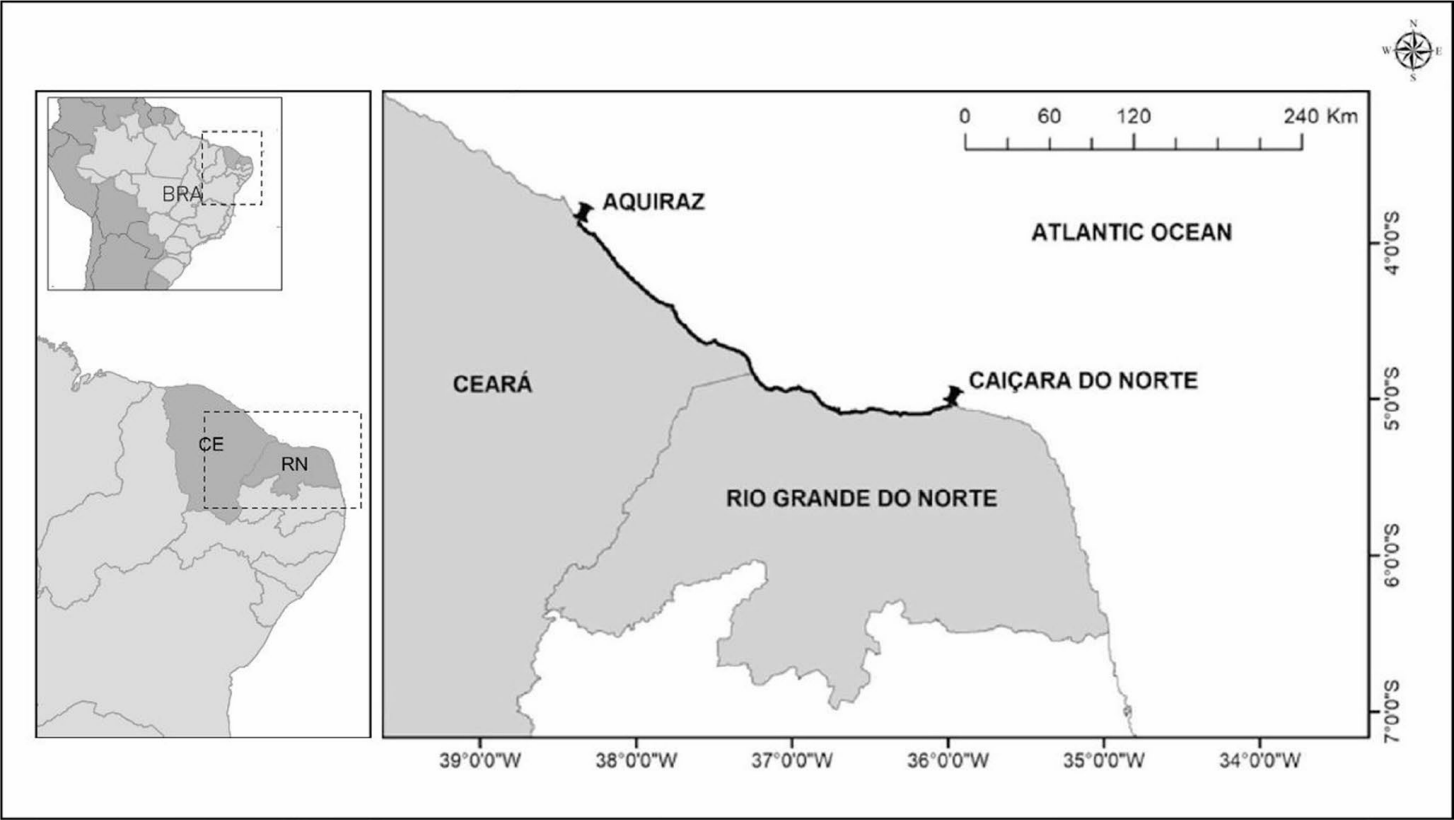
Area monitored by beach monitoring project in the Potiguar Basin: from Aquiraz/Ceará State to Caiçara do Norte/Rio Grande do Norte State. Source: Projeto Cetáceos da Costa Branca - Universidade do Estado do Rio Grande do Norte (PCCB-UERN)

A total of 19 odontocete cetacean specimens, namely *S. guianensis* (*n* = 11; four males, six females, and one individual of undetermined sex) and *P. crassidens* (*n* = 8; four males and four females) were obtained (Table 1), from *Projeto de Monitoramento de Praias* (*Beach Monitoring Project - PMP*) records from the Potiguar Basin. Sampling was conducted by the *Projeto Cetáceos da Costa Branca - Universidade do Estado do Rio Grande do Norte* (PCCB-UERN) (*Cetaceans of the Costa Branca Project – State University of Rio Grande do Norte (PCCB-UERN*) during beach monitoring activities, under SISBIO/ICMBio authorization number 13694-9 between 2013 and yearly from 2015 to 2018, along the coast of the Potiguar Basin. This region is bounded to the northwest by the municipality of Aquiraz (03◦49′ 20.9” S and 38◦24′ 07.8” W) in the state of Ceará, and to the east by the municipality of Caiçara do Norte (05°05′28.6″ S, 36°17′37.9″ W) in the state of Rio Grande do Norte (Fig. 1). The PMP in the Potiguar Basin has been coordinated by PCCB-UERN since 2010, as part of environmental monitoring obligations required by IBAMA in response to oil and gas exploration activities by PETROBRAS (Petróleo Brasileiro S.A.; Agreement number 2500.005657510.2).

**Table 1:** Data on the investigated odontocete cetaceans, including animal code, species, decomposition code, total length in centimeters (cm), life stage and sex

Animal code	Species	Decomposition code	Total length (cm)	Age class	Sex
PC 01	*Pseudorca crassidens*	2	489	Adult	Male
PC 02	*Pseudorca crassidens*	2	510	Adult	Male
PC 03	*Pseudorca crassidens*	2	449	Adult	Female
PC 04	*Pseudorca crassidens*	2	176	Juvenile	Male
PC 05	*Pseudorca crassidens*	2	394	Adult	Female
PC 06	*Pseudorca crassidens*	2	514	Adult	Male
PC 07	*Pseudorca crassidens*	2	422	Adult	Female
PC 08	*Pseudorca crassidens*	4	415	Adult	Female
SG 01	*Sotalia guianensis*	3	175	Adult	Female
SG 02	*Sotalia guianensis*	4	139	Juvenile	Female
SG 03	*Sotalia guianensis*	4	185	Adult	Male
SG 04	*Sotalia guianensis*	4	200	Adult	Male
SG 05	*Sotalia guianensis*	4	*	*	*
SG 06	*Sotalia guianensis*	3	104	Juvenile	Female
SG 07	*Sotalia guianensis*	3	160	Adult	Female
SG 08	*Sotalia guianensis*	2	196	Adult	Female
SG 09	*Sotalia guianensis*	3	112	Juvenile	Female
SG 10	*Sotalia guianensis*	3	174	Juvenile	Male
SG 11	*Sotalia guianensis*	3	127	Juvenile	Male

*Indeterminate

The animals originated from strandings, found in different decomposition stages (codes 2, 3, and 4) [33], or corresponded to live individuals that died during rehabilitation. Briefly, code 2 represents fresh carcasses in good condition with minimal decomposition, code 3 includes moderately decomposed carcasses with preserved organ integrity, and code 4 corresponds to advanced decomposition, with marked tissue degradation. Sex was determined during necropsy, while age classes were established based on total body length and morphological gonad assessment. Individuals whose gonads exhibited histological features indicative of reproductive activity were classified as adults, whereas those lacking such characteristics were considered juvenile [34, 35]. About 0.3 g aliquots of muscle, liver, and kidney samples were collected, stored in polyethylene Ziploc^®^ bags, and kept frozen at -20 °C until analysis.

### Metal(loid) Determinations

Due to limited sample availability, it was not possible to analyze all three tissue types in every individual, although at least one element was assessed in a minimum of one tissue type from each specimen.

Hg analyses were performed according to Bisi et al. [36]. Briefly, 0.3 g (wet weight) of muscle, kidney, and liver were digested with hydrogen peroxide (Merck^®^) and a mixed solution of sulfuric (Merck^®^) and nitric (Merck^®^) acids at a 1:1 ratio. The extracts were heated at 60 °C for two hours in a water bath, followed by the addition of potassium permanganate (KMnO_4_, 5%, Sigma-Aldrich^®^). Total Hg concentrations were determined by cold vapor atomic absorption spectroscopy (CVAAS – Flow Injection Mercury System, FIMS-400, Perkin Elmer). The accuracy and precision of the analytical method was verified using the certified reference material (CRM) DORM-3 (National Research Council – NRC, Canada) and an internally certified material (MAQUA ICM), besides the analysis of analytical blanks and duplicate samples, with a relative percent difference of < 20%. The CRM recoveries were as follows: DORM-3 = 103.08 ± 2.4%, and for MAQUA ICM: MIR-F1 = 108.29 ± 2.0% and MIR-M1 = 100.71 ± 2.87%. The Limit of Detection (LoD) for Hg was 0.000017 µg L⁻¹ and the Limit of Quantification (LOQ) was 0.00077 µg g⁻¹. Concentrations were expressed as µg g⁻¹ wet weight.

The remaining elements (Cu, Cd, Ag, and Se) were determined following Dorneles et al. [37]. Briefly, two aliquots of about 0.3 g of each sample were mixed with 2 mL of nitric acid (HNO₃, 65%, Merck^®^) and left to stand overnight. The solutions were then heated at 60 °C in a water bath for two hours. After cooling, they were transferred to 15 mL tubes and made up with ultrapure water (Milli-Q) to a final volume of 10 mL. Elemental concentrations were determined by electrothermal graphite furnace atomic absorption spectrometry (ZEEnit 650P, Analytic Jena). Palladium nitrate (Merck^®^) and magnesium nitrate (Merck^®^) were used as matrix modifiers.

The accuracy and precision of the analytical methods were verified using certified reference materials (TORT-2 and DOLT-4), with mean recoveries (mean ± standard deviation, %) of 99 ± 7.02 (Cu), 96 ± 7.69 (Cd), 100 ± 6.22 (Ag), and 99 ± 4.95 (Se). Quality control was also performed through the analysis of procedural blanks (< 5.0 × 10⁻³ µg g⁻¹) and sample replicates (coefficient of variation < 25%). The LoDs were 0.00045 (Cu), 0.00025 (Cd), 0.00020 (Ag), and 0.005 µg g⁻¹ (Se), with corresponding LOQs of 0.00149, 0.00083, 0.00066, and 0.0165 µg g⁻¹, respectively. Concentrations were expressed as µg g⁻¹ wet weight.

### Data Analysis

Due to the low number of samples, data were evaluated using descriptive statistics in relation to the entire data set, grouped independent of stranding year, and then further stratified by species, sex, and age class, with no further statistical analyses conducted.

## Results and Discussion

Data concerning the metal(loid) determinations in all investigated individuals are depicted in Table S1. Table 2 presents the descriptive statistics (median, range, mean ± SD, and sample size) for all analyzed elements and tissues in both cetacean species. Given the limited sample sizes for several elements, these statistics are presented for completeness but should be interpreted cautiously.

**Table 2:** Descriptive statistics for element concentrations (µg g⁻¹ wet weight) by species and tissue, including sample size (n), range (min–max), median, and mean ± standard deviation. Values less than the limits of detection (< LoD) were excluded from calculations

Species	Tissue	Element	*n*	Range (min–max) (µg g⁻¹)	Median (µg g⁻¹)	Mean ± SD (µg g⁻¹)
*Pseudorca crassidens*	Liver	Hg	8	2.5–1720	779.2	794.1 ± 540.0
*Pseudorca crassidens*	Muscle	Hg	8	1.0–52.6	31.6	30.2 ± 16.8
*Sotalia guianensis*	Liver	Hg	4	0.01–5.2	0.6	1.60 ± 2.30
*Sotalia guianensis*	Muscle	Hg	11	< LoD–1.1	0.2	0.42 ± 0.39
*Sotalia guianensis*	Kidney	Hg	2	< LoD–3.1	1.55	1.55 ± 2.19
*Sotalia guianensis*	Liver	Cu	2	9.7–56.3	33.0	33.0 ± 33.0
*Sotalia guianensis*	Muscle	Cu	5	0.4–1.7	0.9	1.00 ± 0.49
*Sotalia guianensis*	Kidney	Cu	1	3.8–3.8	3.8	NA
*Sotalia guianensis*	Liver	Cd	2	0.3–0.3	0.3	NA
*Sotalia guianensis*	Kidney	Cd	1	0.2–0.2	0.2	NA
*Sotalia guianensis*	Liver	Ag	2	0.1–0.1	0.1	0.10 ± 0.00
*Sotalia guianensis*	Muscle	Ag	5	0.9–21.0	11.0	11.0 ± 14.32
*Sotalia guianensis*	Kidney	Ag	1	< LoD – 0.07	0.07	NA
*Sotalia guianensis*	Liver	Se	2	0.5–0.8	0.6	0.65 ± 0.21
*Sotalia guianensis*	Muscle	Se	4	< LoD – 0.4	0.4	NA
*Sotalia guianensis*	Kidney	Se	1	3.6–3.6	3.6	NA

*Hg* mercury, *Cu* copper, *Cd* cadmium, *Ag* silver, *Se* selenium, *NA *not applicable

Eight *P. crassidens* individuals (four males and four females, all adults except for one juvenile male), all found stranded in 2013, were analyzed for Hg in both liver and muscle tissues. Hg was detected in all samples. When grouping both sexes, hepatic concentrations ranged from 2.5 to 1,720 µg g⁻¹, with a mean ± standard deviation (SD) of 794.1 ± 556.9 µg g⁻¹, much higher than in muscle, which ranged from 1.0 to 52.6 µg g⁻¹ (30.2 ± 16.7 µg g⁻¹). When separated by sex, females exhibited higher Hg levels in both liver (974.9 ± 679.9 µg g⁻¹) and muscle (33.5 ± 15.9 µg g⁻¹) than males (613.4 ± 417.5 µg g⁻¹ and 26.8 ± 19.3 µg g⁻¹, respectively). This suggests either higher exposure, reduced elimination efficiency, or possible Hg mobilization during physiological processes such as gestation or lactation in females, although this should be interpreted with caution due to the low sample size analyzed herein. One liver sample was also analyzed for Cu, but the concentration was below the LoD.

A total of eleven *S. guianensis* individuals were found stranded, namely four males (two juveniles and two adults), six females (including three juvenile females and three adults), and one of indeterminate sex. Hg concentrations were analyzed in the liver of only four individuals, whereas muscle tissue was analyzed for all eleven animals, and kidney tissue was analyzed in a single individual.

Although the sample size was too limited to allow for statistical comparisons between liver and muscle Hg concentrations, it is noteworthy that the mean concentration in the four liver samples (1.6 ± 2.4 µg g⁻¹) was considerably higher than in the eleven muscle samples (0.4 ± 0.4 µg g⁻¹). This pattern is associated with the liver’s bioaccumulation potential, as it serves as the primary organ for metabolism and detoxification, resulting in greater exposure and accumulation of this metal [38, 39]. However, the Hg detected in the only kidney sample was even higher (3.1 µg g⁻¹), exceeding values observed in both liver and muscle. This may indicate preferential accumulation or retention of Hg in renal tissue, potentially reflecting recent exposure, species-specific excretion mechanisms, or impaired detoxification processes in that individual [39, 40]. Further assessments on a higher number of samples, however, are required to corroborate or refute this hypothesis.

For this species, two liver samples, both from females, one juvenile and one adult, were also analyzed for Ag, Cu, Cd and Se. Regarding Cd, only the juvenile individual presented detectable levels (0.3 µg g⁻¹). The median concentrations for the two samples were 33.0 µg g⁻¹ for Cu, 0.1 µg g⁻¹ for Ag, and 0.6 µg g⁻¹ for Se.

Regarding muscle tissue, five samples were analyzed for Cu, including the same two individuals analyzed for liver, plus three others (comprising one juvenile male, two juvenile females, and two adult females). The mean Cu concentration was 1.0 ± 0.5 µg g⁻¹. These same five samples were also analyzed for Cd, with all values below the LoD. For Ag, three samples were below the LoD while the remaining two yielded a median concentration of 11.0 µg g⁻¹. Selenium was analyzed in four individuals, one juvenile male, two adult females, and one juvenile female, with two samples below the LoD and a median concentration of 0.4.

Regarding kidney samples, two individuals were analyzed for Hg, one adult female and one juvenile male, presenting concentrations of 3.1 and < LoD µg g⁻¹, respectively. These differences in Hg concentrations observed in the kidney samples of the adult female (3.1 µg g⁻¹) and the juvenile male (0.0 µg g⁻¹) may reflect their different age and exposure times, as the adult female may have experienced a longer cumulative exposure to Hg through diet and environmental contact, allowing for greater bioaccumulation over time, whereas the juvenile male likely had limited exposure due to its younger age and shorter life history [8, 40, 41]. However, other factors may also have contributed to this pattern, such as differences in diet, trophic level and foraging areas between individuals, as well as sex-specific detoxification or excretion rates [41]. Sex-related differences may also be associated with variation in Hg demethylation efficiency and in the availability of physiological stores of elements involved in detoxification processes, particularly Se, which plays an important role in Hg sequestration and detoxification [42]. In addition, interindividual differences in tissue-specific element incorporation and retention rates may further contribute to the observed variability. The high concentration in the adult female could also indicate a recent acute exposure, impaired excretion capacity, or mobilization of Hg from other tissues during physiological stress, starvation, or decomposition [40, 43]. As the individuals were not necessarily derived from the same pod, differences in foraging area, prey composition, and local exposure conditions may also have influenced Hg intake and accumulation patterns [44, 45]. Furthermore, although Hg concentrations may remain relatively stable during decomposition [46], other elements under tighter homeostatic regulation may be more susceptible to variation, particularly in animals experiencing starvation or advanced decomposition. These processes were not directly assessed in the present study, but should be considered as possible contributors to variability in tissue concentrations and warrant targeted investigation in future studies. The adult female was also analyzed for Cu, Cd, Ag, and Se, with concentrations of 3.8, 0.2, 0.07, and 3.6 µg g⁻¹, respectively.

The different metal(loid) patterns detected in the coastal *S. guianensis* and the oceanic *P. crassidens* likely reflect distinct ecological and physiological factors associated to their habitat use and life histories. Oceanic odontocetes, such as *P. crassidens*, generally feed on larger, higher-trophic-level prey, including sizable fish, cephalopods and rarely, other cetaceans [32], which tend to occupy upper marine food web positions and often have longer lifespans, accumulating greater metal(loid) concentrations through biomagnification processes [31, 32]. In contrast, coastal dolphins like *S. Guianensis* present a more diverse diet comprised mainly of smaller teleost fish and invertebrates such as crustaceans and cephalopods, which generally occupy lower trophic levels and have shorter lifespans [25–27]. This dietary variation may result in lower exposure to accumulated metal(loid)s. Additionally, coastal habitats can have differing contaminant sources and dynamics compared to offshore environments [47–49], further influencing the contaminant profiles found in these species. Consequently, numerous ecological variables besides biological and chemical factors, including differences in prey size, trophic level, and habitat contamination patterns may also collectively contribute to the observed differences in metal(loid) concentrations between oceanic and coastal odontocetes.

Previous studies have reported similarly high hepatic Hg concentrations in oceanic odontocetes, reinforcing the influence of trophic position and prey preferences on metal bioaccumulation. For example, *P. crassidens* stranded in the Atlantic Ocean exhibited hepatic Hg levels exceeding 1,000 µg g⁻¹ wet weight, values comparable to those recorded in the present study [8]. In contrast, *S. guianensis* from coastal areas of Southeastern Brazil typically presents hepatic Hg concentrations below 5 µg g⁻¹ wet weight, averaging 1.0 µg g⁻¹ [41], consistent with the lower values observed in our samples. This agreement with previous literature supports the interpretation that dietary differences associated with habitat are a key factor underlying contaminant profiles.

From an ecotoxicological perspective, the high hepatic Hg levels detected in *P. crassidens* are noteworthy, as adverse effects in marine mammals, including neurological impairment, immunosuppression, and reduced reproductive success [15, 50], have been reported alongside concentrations as low as 100 µg g⁻¹ wet weight, ranging up to 1000 µg g⁻¹. However, toxicity thresholds can vary considerably among species, and caution is warranted when interpreting these values, as thresholds established for one tissue type may not be directly applicable to others. In *P. crassidens*, hepatic Hg concentrations reached up to 1,720 µg g⁻¹, markedly higher than those reported in bottlenose dolphins (*Tursiops truncatus*) by Rawson et al. [51], which did not exceed 443 µg g⁻¹. Although *T. truncatus* is also considered an oceanic species, the comparatively higher concentrations observed in *P. crassidens* may reflect its ability to exploit deeper waters, where He exposure is potentially greater, particularly in the form of methylmercury (MeHg), the most toxic form [52]. In *S. guianensis*, despite lower overall metal(loid) levels, the detection of Cd and Ag in some individuals, albeit at low levels, indicates potential exposure to localized anthropogenic sources such as industrial effluents and port activities, highlighting the need for continued monitoring.

It is important to acknowledge that the limited number of analyzed specimens for certain metals and tissues constrains robust statistical comparisons and the ability to fully assess interindividual variability. Nevertheless, the data presented herein provide a valuable baseline for the Potiguar Basin, as scarce ecotoxicological information on marine mammals from this area is available. Future research should aim to increase sample size, incorporate stable isotope analyses to refine trophic position estimates, and investigate potential sources of contaminants in both coastal and oceanic habitats, to clarify exposure pathways, assess temporal trends, and better understand the implications of metal(loid) concentrations for the health and conservation of odontocete populations.

## Conclusion

The findings reported herein revealed differences in metal(loid) profiles between the analyzed two species, with *P. crassidens* exhibiting higher total Hg concentrations, particularly in hepatic tissue, likely reflecting its oceanic distribution, higher trophic position, and consumption of long-lived prey species prone to greater biomagnification. In contrast, *S. guianensis* exhibited lower concentrations for most elements, consistent with its coastal feeding habits on smaller, lower-trophic-level prey. These findings highlight the influence of habitat use, trophic ecology, and life history on metal(loid) bioaccumulation in odontocetes, reinforcing the importance of considering ecological context in marine ecotoxicological assessments. From an ecotoxicological standpoint, the results indicate that the two species may differ in their exposure pathways and relative susceptibility to metal(loid) accumulation, which may aid in guiding future monitoring and conservation efforts. Nevertheless, more in-depth interpretations are precluded due to the limited sample size reported herein.

## Supplementary Information

Below is the link to the electronic supplementary material.

Supplementary Material 1

## Data Availability

No datasets were generated or analysed during the current study.

## References

[1] Peters KJ, Bury SJ, Hinton B, Betty EL, Casano-Bally D, Parra GJ, Stockin KA (2022) Too close for comfort? isotopic niche segregation in New Zealand’s odontocetes. Biology 11:1179. 10.3390/biology11081179 36009806 10.3390/biology11081179 PMC9405429

[2] Ricci P, Serpetti N, Cascione D, Cipriano G, D’Onghia G, De Padova D, Fanizza C, Ingrosso M, Carlucci R (2023) Investigating fishery and climate change effects on the conservation status of odontocetes in the Northern Ionian Sea (Central Mediterranean Sea). Ecol Model 485:110500. 10.1016/j.ecolmodel.2023.110500

[3] Bossart GD (2011) Marine mammals as sentinel species for oceans and human health. Vet Pathol 48:676–690. 10.1177/0300985810388525 21160025 10.1177/0300985810388525

[4] Dool T, Bosker T (2022) Predicted microplastic uptake through trophic transfer by the short-beaked common dolphin (*Delphinus delphis*) and common bottlenose dolphin (*Tursiops truncatus*) in the Northeast Atlantic Ocean and Mediterranean Sea. Mar Pollut Bull 180:113745. 10.1016/j.marpolbul.2022.113745 35653906 10.1016/j.marpolbul.2022.113745

[5] Padula AD, Machado R, Milmann L, De León MC, Gana JCM, Wickert JC, Argañaraz ME, Bastida RO, Rodríguez DH, Denuncio PE (2023) Marine debris ingestion by odontocete species from the Southwest Atlantic Ocean: absence also matter. Mar Pollut Bull 186:114486. 10.1016/j.marpolbul.2022.114486

[6] Radaelli E, Palladino G, Nanetti E, Scicchitano D, Rampelli S, Airoldi S, Candela M, Marangi M (2024) Meta-analysis of the Cetacea gut microbiome: diversity, co-evolution, and interaction with the anthropogenic pathobiome. Sci Total Environ 932:172943. 10.1016/j.scitotenv.2024.172943 38714258 10.1016/j.scitotenv.2024.172943

[7] Ali H, Khan E, Ilahi I (2019) Environmental chemistry and ecotoxicology of hazardous heavy metals: Environmental persistence, toxicity, and bioaccumulation. J Chem 2019:1–14. 10.1155/2019/6730305

[8] Page A, Hay C, Marks W, Bennett B, Gribble MO, Durden WN, Stolen M, Jablonski T, Gordon N, Kolkmeyer T, Jiang M, Pegg N, Brown H, Burton S (2024) Trace element bioaccumulation, tissue distribution, and elimination in odontocetes stranded in Florida and Georgia, USA over a 15-year period (2007–2021). Heliyon 10:25552. 10.1016/j.heliyon.2024.e25552 10.1016/j.heliyon.2024.e25552 PMC10865268 38356552

[9] Endo T, Haraguchi K, Sakata M (2002) Mercury and selenium concentrations in the internal organs of toothed whales and dolphins marketed for human consumption in Japan. Sci Total Environ 300(1–3):15–22. 10.1016/s0048-9697(02)00137-7 12685467 10.1016/s0048-9697(02)00137-7

[10] Aziz KHH, Mustafa FS, Omer KM, Hama S, Hamarawf RF, Rahman KO (2023) Heavy metal pollution in the aquatic environment: efficient and low-cost removal approaches to eliminate their toxicity: a review. RSC Adv 13:17595–17610. 10.1039/d3ra00723e 37312989 10.1039/d3ra00723e PMC10258679

[11] Reed LA, McFee WE, Pennington PL, Wirth EF, Fulton MH (2015) A survey of trace element distribution in tissues of the dwarf sperm whale (*Kogia sima*) stranded along the South Carolina coast from 1990–2011. Mar Pollut Bull 100:501–506. 10.1016/j.marpolbul.2015.09.005 26386505 10.1016/j.marpolbul.2015.09.005

[12] Caurant F, Navarro M, Amiard JC (1996) Mercury in pilot whales: possible limits to the detoxification process. Sci Total Environ 186(1–2):95–104. 10.1016/0048-9697(96)05087-5 8685711 10.1016/0048-9697(96)05087-5

[13] Arteel GE, Sies H (2001) The biochemistry of selenium and the glutathione system. Environ Toxicol Pharmacol 10(4):153–158. 10.1016/s1382-6689(01)00078-3 21782571 10.1016/s1382-6689(01)00078-3

[14] Stavros HC, Stolen M, Durden WN, McFee W, Bossart GD, Fair PA (2011) Correlation and toxicological inference of trace elements in tissues from stranded and free-ranging bottlenose dolphins (*Tursiops truncatus*). Chemosphere 82:1649–1661. 10.1016/j.chemosphere.2010.11.019 21126751 10.1016/j.chemosphere.2010.11.019

[15] López-Berenguer G, Peñalver J, Martínez-López E (2019) A critical review about neurotoxic effects in marine mammals of mercury and other trace elements. Chemosphere 246:125688. 10.1016/j.chemosphere.2019.125688 31896013 10.1016/j.chemosphere.2019.125688

[16] Seguel M, George RC, Maboni G, Sanchez S, Page-Karjian A, Wirth E, McFee W, Gottdenker NL (2020) Pathologic findings and causes of death in bottlenose dolphins *Tursiops truncatus* stranded along the Georgia coast, USA (2007–2013). Dis Aquat Org 141:25–38. 10.3354/dao03509 10.3354/dao03509 32940248

[17] Meirelles C, Monteiro-Neto C, Martins AMA, Costa AF, Barros H, De Oliveira Alves MD (2009) Cetacean strandings on the coast of Ceará, north-eastern Brazil (1992–2005). Mar Biol Assoc UK 89:1083–1090. 10.1017/S0025315409002215

[18] Lima SA, Lima MA, Attademo FLN, Oliveira REM, Ambrosio GML, Silva FJL (2021) Diversidade e distribuição espacial de mamíferos marinhos no Rio Grande do Norte (Brasil). [Diversity and spatial distribution of marine mammals in Rio Grande do Norte (Brazil)]. Meio Ambiente 3:46–57

[19] Lemos LS, De Moura JF, Hauser-Davis RA, De Campos RC, Siciliano S (2013) Small cetaceans found stranded or accidentally captured in southeastern Brazil: bioindicators of essential and non-essential trace elements in the environment. Ecotoxicol Environ Saf 97:166–175. 10.1016/j.ecoenv.2013.07.025 23993648 10.1016/j.ecoenv.2013.07.025

[20] Silva VS, Skueresky N, Lopes F, Koch TK, Ott PH, Siciliano S, Barreto AS, Secchi ER, De Meirelles ACO, Carvalho VL, Borges JCG, Danilewicz D, Farro APC, Barbosa LA, Martins SJ Jr., Domit C, Serrano I, Da Silva T, Trinca C, Marmontel M, Emin-Lima NR, Vliati VH, Eizirik E, De Oliveira LR (2021) Integrating morphology and DNA barcoding to assess cetacean diversity in Brazil. Mammal Res 66:349–369. 10.1007/s13364-021-00555-w

[21] Maia RP, Bezerra FHR (2015) Potiguar Basin: diversity of landscapes in the brazilian equatorial margin. In: Vieira B, Salgado A, Santos L (eds) Landscapes and Landforms of Brazil. World Geomorphological Landscapes. Springer, Dordrecht, pp 147–156. 10.1007/978-94-017-8023-0_13

[22] Farias DSD, Rossi S, Costa Bomfim A, Fragoso ABL, Santos-Neto EB, Lima Silva FJ, Lailson-Brito J, Navoni JA, Gavilan AS, Amaral VS (2022) Bioaccumulation of total mercury, copper, cadmium, silver, and selenium in green turtles (*Chelonia mydas*) stranded along the Potiguar Basin, northeastern Brazil. Chemosphere 299:134331. 10.1016/j.chemosphere.2022.134331 35339524 10.1016/j.chemosphere.2022.134331

[23] Azevedo AF, Oliveira AM, Viana SC, Sluys MV (2007) Habitat use by marine tucuxis (*Sotalia guianensis*) (Cetacea: Delphinidae) in Guanabara Bay, south-eastern Brazil. J Mar Biol Assoc UK 87:201–205. 10.1017/S0025315407054422

[24] Flach L, Flach PA, Chiarello AG (2008) Aspects of behavioral ecology of *Sotalia guianensis* in Sepetiba Bay, southeast Brazil. Mar Mammal Sci 24:503–515. 10.1111/J.1748-7692.2008.00198.X

[25] Pansard KCA, Gurgel HCB, Araújo Andrade LC, Yamamoto ME (2011) Feeding ecology of the estuarine dolphin (*Sotalia guianensis*) on the coast of Rio Grande do Norte, Brazil. Mar Mammal Sci 27:673–687. 10.1111/J.1748-7692.2010.00436.X

[26] Rodrigues VLA, Wedekin LL, Marcondes MCC, Barbosa L, Farro APC (2020) Diet and foraging opportunism of the Guiana dolphin (*Sotalia guianensis*) in the Abrolhos Bank, Brazil. Mar Mammal Sci 36:436–450. 10.1111/mms.12656

[27] Vital N, Guari EB, Bisi T, Flach L, Júnior JLB, Freitas Azevedo A (2024) Feeding habits of the Guiana dolphin, *Sotalia guianensis*, (Van Bénéden, 1864) (Cetacea: Delphinidae) in Sepetiba and Ilha Grande bays, southeastern Brazil. Reg Stud Mar Sci 73:103446. 10.1016/j.rsma.2024.103446

[28] Baird RW, Gorgone AM, McSweeney DJ, Webster DL, Salden DR, Deakos MH, Ligon AD, Schorr GS, Barlow J, Mahaffy SD (2008) False killer whales (*Pseudorca crassidens*) around the main Hawaiian Islands: Long-term site fidelity, inter-island movements, and association patterns. Mar Mammal Sci 24:591–612. 10.1111/J.1748-7692.2008.00200.X

[29] Palmer C, Martien KK, Raudino H, Robertson KM, Withers A, Withers E, Risk R, Cooper D, D’Cruz E, Jungine E, Barrow D, Cuff N, Lane A, Keynes D, Waples K, Malpartida A, Banks S (2023) Evidence of resident coastal population(s) of false killer whales (*Pseudorca crassidens*) in northern Australian waters. Front Commun 9:10676600. 10.3389/fmars.2022.1067660

[30] Zaeschmar JR, Dwyer SL, Stockin KA (2013) Rare observations of false killer whales (*Pseudorca crassidens*) cooperatively feeding with common bottlenose dolphins (*Tursiops truncatus*) in the Hauraki Gulf, New Zealand. Mar Mammal Sci 29:555–562. 10.1111/J.1748-7692.2012.00582.X

[31] Ortega-Ortiz CD, Elorriaga-Verplancken FR, Olivos-Otriz A, Liñán-Cabello MA, Vargas-Bravo MH (2014) Insights into the feeding habits of false killer whales (*Pseudorca crassidens*) in the Mexican Central Pacific. Aquat Mamm 40:386–393. 10.1578/AM.40.4.2014.386

[32] Bianucci G, Geisler JH, Citron S, Collareta A (2022) The origins of the killer whale ecomorph. Curr Biol 32:1843–1851. 10.1016/j.cub.2022.02.041. .e2 35259339 10.1016/j.cub.2022.02.041

[33] Geraci JR, Lounsbury VJ (1993) Marine mammals ashore: a field guide for strandings. Texas A&M University Sea Grant College Program. Available at: https://repository.library.noaa.gov/view/noaa/39015

[34] Ferrero RC, Walker WA (1993) Growth and reproduction of the northern right whale dolphin, *Lissodelphis borealis*, in the offshore waters of the North Pacific Ocean. Can J Zool 71:2335–2344. 10.1139/z93-328

[35] Sorensen TB, Kinze CC (1994) Reproduction and reproductive seasonality in Danish harbour porpoises, *Phocoena phocoena*. Ophelia 39(3):159–176. 10.1080/00785326.1994.10429541

[36] Bisi TL, Lepoint G, Azevedo AF, Dorneles PR, Flach L, Das K, Malm O, Lailson-Brito J (2012) Trophic relationships and mercury biomagnification in Brazilian tropical coastal food webs. Ecol Indicat 18:291–302. 10.1016/j.ecolind.2011.11.015

[37] Dorneles PR, Lailson-Brit J, Secchi ER, Bassoi M, Lozinsky CPC, Torres JPM, Malm O (2007) Cadmium concentrations in franciscana dolphin (*Pontoporia blainvillei*) from south brazilian coast. Braz J Oceanogr 55:179–186

[38] Monteiro-Neto C, Itavo RV, De Souza Moraes LE (2003) Concentrations of heavy metals in *Sotalia fluviatilis* (Cetacea: Delphinidae) off the coast of Ceará, northeast Brazil. Environ Pollut 123:319–324. 10.1016/S0269-7491(02)00371-8 12628211 10.1016/s0269-7491(02)00371-8

[39] McCormack MA, Fielding R, Kiszka JJ, Paz V, Jackson BE, Bergfelt DR, Dutton J (2019) Mercury and selenium concentrations, and selenium: mercury molar ratios in small cetaceans taken off St. Vincent, West Indies. Environ Res 181:108908. 10.1016/j.envres.2019.108908 31759648 10.1016/j.envres.2019.108908

[40] Kershaw JL, Hall AJ (2019) Mercury in cetaceans: exposure, bioaccumulation and toxicity. Sci Total Environ 694:133683. 10.1016/j.scitotenv.2019.133683 31394330 10.1016/j.scitotenv.2019.133683

[41] Moura JF, Souza Hacon S, Veja CM, Hauser-Davis RA, Campos RC, Siciliano S (2012) Guiana dolphins (*Sotalia guianensis*, Van Benédén 1864) as indicators of the bioaccumulation of total mercury along the Coast of Rio de Janeiro State, Southeastern Brazil. Bull Environ Contam Toxicol 88:54–59. 10.1007/s00128-011-0448-z 22057227 10.1007/s00128-011-0448-z

[42] Palmisano F, Cardellicchio N, Zambonin PG (1995) Speciation of mercury in dolphin liver: a two-stage mechanism for the demethylation accumulation process and role of selenium. Mar Environ Res 40(2):109–121. 10.1016/0141-1136(94)00142-C

[43] Frodello JP, Roméo M, Viale D (2000) Distribution of mercury in the organs and tissues of five toothed-whale species of the Mediterranean. Environ Pollut 108(3):447–452. 10.1016/S0269-7491(99)00221-3 15092940 10.1016/s0269-7491(99)00221-3

[44] Choy C, Popp B, Kaneko J, Drazen J (2009) The influence of depth on mercury levels in pelagic fishes and their prey. Proc Natl Acad Sci 106(33):13865–13869. 10.1073/pnas.0900711106 19666614 10.1073/pnas.0900711106 PMC2728986

[45] Croizier GL, Sonke JE, Lorrain A, Renedo M, Hoyos-Padilla M, Santana-Morales O, Meyer L, Huveneers C, Butcher P, Amezcua-Martínez F, Point D (2021) Foraging plasticity diversifies mercury exposure sources and bioaccumulation patterns in the world’s largest predatory fish. Hazard Mater 425:127956. 10.1016/j.jhazmat.2021.127956 10.1016/j.jhazmat.2021.127956 34986563

[46] Siebert U, Joiris C, Holsbeek L, Benke H, Failing K, Frese K, Petzinger E (1999) Potential relation between mercury concentrations and necropsy findings in cetaceans from sGerman waters of the north and baltic seas. Mar Pollut Bull 38(4):285–295. 10.1016/s0025-326x(98)00147-7

[47] Li C, Wang H, Liao X, Xiao R, Liu K, Bai J, He Q (2021) Heavy metal pollution in coastal wetlands: a systematic review of studies globally over the past three decades. J Hazard Mater 424:127312. 10.1016/j.jhazmat.2021.127312 34600393 10.1016/j.jhazmat.2021.127312

[48] Liu M, Zhang Q, Maavara T, Liu S, Wang X, Raymond PA (2021) Rivers as the largest source of mercury to coastal oceans worldwide. Nat Geosci 14:672–677. 10.1038/s41561-021-00793-2

[49] Fioramonti NE, Guevara SR, Becker YA, Riccialdelli L (2022) Mercury transfer in coastal and oceanic food webs from the Southwest Atlantic Ocean. Mar Pollut Bull 175:113365. 10.1016/j.marpolbul.2022.113365 35114547 10.1016/j.marpolbul.2022.113365

[50] Cáceres-Saez I, Haro D, Blank O, Lobo AA, Dougnac C, Arredondo C, Cappozzo HL, Guevara SR (2018) High status of mercury and selenium in false killer whales (*Pseudorca crassidens*, Owen 1846) stranded on Southern South America: a possible toxicological concern? Chemosphere 199:637–646. 10.1016/j.chemosphere.2018.02.046 29462769 10.1016/j.chemosphere.2018.02.046

[51] Rawson AJ, Patton GW, Hofmann S, Pietra GG, Johns L (1993) Liver abnormalities associated with chronic mercury accumulation in stranded atlantic bottlenosed dolphins. Ecotoxicol Environ Saf 25:41–47. 10.1006/eesa.1993.1005 7682917 10.1006/eesa.1993.1005

[52] Romero-Romero S, García-Ordiales E, Roqueñí N, Acuña JL (2022) Increase of mercury and methylmercury levels with depth in a fish assemblage. Chemosphere 292:133445. 10.1016/j.chemosphere.2021.13344 34968522 10.1016/j.chemosphere.2021.133445

