# Supplementary material for: Metal(loid) Concentrations in Coastal (Sotalia guianensis) and Oceanic (Pseudorca crassidens) Odontocete Cetaceans Stranded Along the Northeastern Brazilian Equatorial Margin
Source: Biol Trace Elem Res. 2026 May 5;204(9):6643–51. doi: 10.1007/s12011-026-05137-y (PMC13437707; doi:10.1007/s12011-026-05137-y)
Supplement: Supplementary file 1 [file 12011_2026_5137_MOESM1_ESM.docx]

**Supplemental Material**

Supplement to “Metal(loid) concentrations in coastal (*Sotalia guianensis*) and oceanic (*Pseudorca crassidens*) odontocete cetaceans stranded along the northeastern Brazilian equatorial margin”

Table S1. Data on the investigated odontocete species (including animal code, life stage and sex) and metal(loid) concentrations (µg g⁻¹ wet weight) in liver, muscle, and kidneys.

| **Animal code** | **Species** | **Age class** | **Sex** | **Liver** | | | | | **Muscle** | | | | | **Kidney** | | | | |
| --- | --- | --- | --- | --- | --- | --- | --- | --- | --- | --- | --- | --- | --- | --- | --- | --- | --- | --- |
|  |  |  |  | **Hg** | **Cu** | **Cd** | **Ag** | **Se** | **Hg** | **Cu** | **Cd** | **Ag** | **Se** | **Hg** | **Cu** | **Cd** | **Ag** | **Se** |
| PC 01 | *Pseudorca crassidens* | Adult | Male | 687.7 | <LoD | NA | NA | NA | 34.1 | NA | NA | NA | NA | NA | NA | NA | NA | NA |
| PC 02 | *Pseudorca crassidens* | Adult | Male | 892.6 | NA | NA | NA | NA | 25.3 | NA | NA | NA | NA | NA | NA | NA | NA | NA |
| PC 03 | *Pseudorca crassidens* | Adult | Female | 1360.2 | NA | NA | NA | NA | 52.6 | NA | NA | NA | NA | NA | NA | NA | NA | NA |
| PC 04 | *Pseudorca crassidens* | Juvenile | Male | 2.5 | NA | NA | NA | NA | 1 | NA | NA | NA | NA | NA | NA | NA | NA | NA |
| PC 05 | *Pseudorca crassidens* | Adult | Female | 260.8 | NA | NA | NA | NA | 14.5 | NA | NA | NA | NA | NA | NA | NA | NA | NA |
| PC 06 | *Pseudorca crassidens* | Adult | Male | 870.6 | NA | NA | NA | NA | 46.9 | NA | NA | NA | NA | NA | NA | NA | NA | NA |
| PC 07 | *Pseudorca crassidens* | Adult | Female | 1720 | NA | NA | NA | NA | 37.9 | NA | NA | NA | NA | NA | NA | NA | NA | NA |
| PC 08 | *Pseudorca crassidens* | Adult | Female | 558.4 | NA | NA | NA | NA | 29.1 | NA | NA | NA | NA | NA | NA | NA | NA | NA |
| SG 01 | *Sotalia guianensis* | Adult | Female | NA | NA | NA | NA | NA | 0.38 | NA | NA | NA | NA | NA | NA | NA | NA | NA |
| SG 02 | *Sotalia guianensis* | Juvenile | Female | NA | NA | NA | NA | NA | 0.8 | NA | NA | NA | NA | NA | NA | NA | NA | NA |
| SG 03 | *Sotalia guianensis* | Adult | Male | 5.2 | NA | NA | NA | NA | 0.8 | NA | NA | NA | NA | NA | NA | NA | NA | NA |
| SG 04 | *Sotalia guianensis* | Adult | Male | NA | NA | NA | NA | NA | 0.1 | NA | NA | NA | NA | NA | NA | NA | NA | NA |
| SG 05 | *Sotalia guianensis* | * | * | NA | NA | NA | NA | NA | 0.9 | NA | NA | NA | NA | NA | NA | NA | NA | NA |
| SG 06 | *Sotalia guianensis* | Juvenile | Female | 0.5 | 9.7 | 0.3 | 0.1 | 0.8 | 0.2 | 0.9 | <LoD | <LoD | <LoD | NA | NA | NA | NA | NA |
| SG 07 | *Sotalia guianensis* | Adult | Female | 0.7 | 56.3 | <LoD | 0.1 | 0.5 | 0.2 | 1.7 | <LoD | 0.9 | 0.4 | NA | NA | NA | NA | NA |
| SG 08 | *Sotalia guianensis* | Adult | Female | NA | NA | NA | NA | NA | 1.1 | 0.4 | <LoD | <LoD | 0.4 | 3.1 | 3.8 | 0.2 | 0.07 | 3.6 |
| SG 09 | *Sotalia guianensis* | Juvenile | Female | NA | NA | NA | NA | NA | 0.06 | 0.7 | <LoD | <LoD | NA | NA | NA | NA | NA | NA |
| SG 10 | *Sotalia guianensis* | Juvenile | Male | 0.01 | NA | NA | NA | NA | 0 | NA | NA | NA | NA | 0 | NA | NA | NA | NA |
| SG 11 | *Sotalia guianensis* | Juvenile | Male | NA | NA | NA | NA | NA | 0.1 | 1.3 | <LoD | 21.2 | <LoD | NA | NA | NA | NA | NA |

NA = Not analyzed; LoD = Limit of Detection; *Indeterminate.
